# Two Deoxythymidine Triphosphate Synthesis-Related Genes Regulate Obligate Symbiont Density and Reproduction in the Whitefly *Bemisia tabaci* MED

**DOI:** 10.3389/fphys.2020.574749

**Published:** 2021-02-24

**Authors:** Zezhong Yang, Cheng Gong, Yuan Hu, Jie Zhong, Jixing Xia, Wen Xie, Xin Yang, Zhaojiang Guo, Shaoli Wang, Qingjun Wu, Youjun Zhang

**Affiliations:** Department of Plant Protection, Institute of Vegetables and Flowers, Chinese Academy of Agricultural Sciences, Beijing, China

**Keywords:** *Bemisia tabaci* MED, candidatus portiera aleyrodidarum, deoxythymidine triphosphate, RNAi, pest control

## Abstract

Deoxythymidine triphosphate (dTTP) is essential for DNA synthesis and cellular growth in all organisms. Here, genetic capacity analysis of the pyrimidine pathway in insects and their symbionts revealed that dTTP is a kind of metabolic input in several host insect/obligate symbiont symbiosis systems, including *Bemisia tabaci* MED/*Candidatus Portiera aleyrodidarum* (hereafter *Portiera*). As such, the roles of dTTP on both sides of the symbiosis system were investigated in *B. tabaci* MED/*Portiera*. Dietary RNA interference (RNAi) showed that suppressing dTTP production significantly reduced the density of *Portiera*, significantly repressed the expression levels of horizontally transferred essential amino acid (EAA) synthesis-related genes, and significantly decreased the reproduction of *B. tabaci* MED adults as well as the hatchability of their offspring. Our results revealed the regulatory role of dTTP in *B. tabaci* MED/*Portiera* and showed that dTTP synthesis-related genes could be potential targets for controlling *B. tabaci* as well as other sucking pests.

## Introduction

Sucking insects harbor intracellular symbionts such as obligate symbionts and facultative symbionts, which affect the fitness of their host in many ways (Baumann, 2005; Ferrari and Vavre, 2011; Douglas, 2015). These intracellular symbionts provide essential nutrients for their hosts (Zientz et al., 2004; Douglas, 2015), protect their hosts from natural enemies and stress (Oliver et al., 2003; Oliver and Higashi, 2019; Zhang et al., 2019) and suppress the plant defense of their hosts (Frago et al., 2012). Because obligate symbionts have reduced genomes and lose many functional genes (McCutcheon and Moran, 2011; Moran and Bennett, 2014), they generally require their host to provide metabolic inputs (Martinez-Cano et al., 2014; Wilson and Duncan, 2015). To date, metabolic inputs supplied by hosts have been shown to directly participate in the metabolism of obligate symbionts. For example, *Portiera* relies on host-provided phosphoenolpyruvic acid, erythrose-4P, pyruvate and 5-phosphoribosyl diphosphate for synthesizing EAA (Xie et al., 2018). Similar trends have also been identified in other complementary EAA biosynthesis pathways in the *Acyrthosiphon pisum*/*Buchnera aphidicola* (hereafter *Buchnera*) symbiosis system (Wilson et al., 2010), *Pachypsylla venusta*/*Carsonella ruddii* symbiosis system (Sloan et al., 2014), *Planococcus citri*/*Tremblaya princeps* symbiosis system (Husnik et al., 2013), and *Nilaparvata lugens*/yeast-like symbiont symbiosis system (Xue et al., 2014). In addition to directly participating in the metabolism of obligate symbionts, the regulatory role of metabolic inputs has also been revealed. A previous study reported that cystathionine input determined methionine production in the *A. pisum*/*Buchnera* symbiosis system (Russell et al., 2014).

The compound dTTP, which is produced by *thymidylate synthase via de novo* biosynthesis or *thymidine kinase via* pyrimidine salvage, is a kind of pyrimidine nucleotide and is essential for housed bacteria as well as bacterial pathogens (Samant et al., 2008; Hashimoto et al., 2012; Wilson et al., 2012; Leija et al., 2016; Yang et al., 2017). As free-living bacteria encode all genes involved in dTTP synthesis, dTTP is not frequently considered an essential exogenous nutrient in bacteria (Turnbough and Switzer, 2008). However, several obligate symbionts of insects have been reported to be unable to produce dTTP (Zientz et al., 2004; Degnan et al., 2011; Moran and Bennett, 2014; Waterworth et al., 2020). As there is a great demand for dTTP throughout the life cycle of symbionts, these findings indicated that dTTP is likely a kind of metabolic input in several insect/obligate symbiont symbiosis systems. Among dTTP synthetic genes, it has been shown that *thymidylate synthase* and *thymidine kinase* served key roles (Carter, 1956; Hartman and Buchanan, 1959). Considering that functions of the two genes have been revealed by previous publications which indicated the two genes are critical for the growth of bacterial pathogens (Fivian-Hughes et al., 2012; Hashimoto et al., 2012; Wilson et al., 2012; Leija et al., 2016; Yang et al., 2017), thymidylate synthase and thymidine kinase have been put forward as molecular targets for bacterial pathogens in recent years (Choi et al., 2016; Leija et al., 2016).

*Bemisia tabaci*, which comprises a number of cryptic species (De Barro et al., 2011) and causes huge economic lost (Liu et al., 2012; Ning et al., 2015; Pan et al., 2015), harbors an obligate symbiont, *Portiera*, as well complexes of facultative symbionts (Chiel et al., 2007; Pan et al., 2012b). To date, the genomes of several *B. tabaci* cryptic species and their symbionts have been sequenced (Jiang et al., 2012; Rao et al., 2012a,b; Santos-Garcia et al., 2012, 2015; Chen et al., 2016, 2019; Xie et al., 2017, 2018). Additionally, several horizontally transferred EAA biosynthesis-related genes have been identified in *B. tabaci* (Luan et al., 2015; Chen et al., 2016; Xie et al., 2018). In terms of compensating for *Portiera* gene loss in EAA biosynthesis (Luan et al., 2015; Xie et al., 2018), those genes provided an easy route for evaluating EAA biosynthetic levels in obligate symbiont. As EAA biosynthesis is the majority metabolism in *Portiera* (Baumann, 2005; Luan et al., 2015; Xie et al., 2018), those HTGs provided an easy route for evaluating metabolism of *Portiera.*

The genomes of many sucking insects and those of their obligate symbionts are currently available, so global views on various metabolic interactions, such as metabolic inputs, in insect/obligate symbiont symbiosis systems are feasible (Douglas, 2018). In this study, genetic capacity analysis of the pyrimidine pathway, especially dTTP, was first performed in several obligate symbionts and host insects. The *B. tabaci* MED/*Portiera* symbiosis system was then applied to investigate the roles of dTTP supplied on both sides of the symbiosis system. Two dTTP synthesis-related genes of *B. tabaci* MED, *thymidylate synthase* (*BtTS*) and *thymidine kinase* (*BtTK*), were identified and cloned. By the silencing of these two genes, the influences of blocking dTTP production on *Portiera* density and EAA biosynthesis were investigated. Furthermore, the possible involvement of these two genes in *B. tabaci* MED management was also investigated.

## Materials and Methods

### Genetic Capacity Analysis of the Pyrimidine Pathway in Insects and Their Obligate Symbionts

For genetic capacity analysis of the pyrimidine pathway, genes that are involved in these processes were identified as follows. Initially, predicted protein sets of *B. tabaci*, *Portiera*, and other selected species (detailed information of which is shown in Supplementary Tables 1, 2) were annotated by KOBAS 2.0 (Wu et al., 2006). Genes that are part of the pyrimidine pathway were then selected. In addition, the sequences of known pyrimidine pathway-related proteins of *A. pisum*, *Apis mellifera*, *Bombyx mori*, *Aedes aegypti*, *Drosophila melanogaster*, *Escherichia coli* K-12 MG1655, *Bacillus subtilis* subsp. subtilis 168 and *Staphylococcus aureus* subsp. aureus N315 (MRSA/VSSA) were also used as queries to search against both predicted proteomes (BLASTP, e-value cutoff of 1E-3) and genome assembly sequences (TBLASTN, e-value cutoff of 1E-5) of selected species (Camacho et al., 2009). In the end, all hits were pooled and verified by searching *via* BLAST against the nr (NCBI) database.

### Insect Strain

*Bemisia tabaci* MED was collected from fields in Beijing, China, in 2009. Since then, the *B. tabaci* MED insects have been reared on cotton (*Gossypium herbaceum* L. cv. Zhongmian 49) in a glasshouse (Pan et al., 2012a). Every three generations, the purity of the strain was monitored as previously described (Chu et al., 2010).

### RNA Isolation, Gene Cloning and Quantitative Real-Time PCR Assays

Total RNA of each sample was extracted by using TRIzol reagent (Invitrogen, Carlsbad, CA, United States) following the manufacturer’s instructions. The integrity of the extracted RNA was checked with 1% agarose gel electrophoresis. The quality of the extracted RNA was then measured *via* a NanoDrop 2000 instrument (Thermo Scientific, Wilmington, DE, United States), and the concentration of each RNA sample was adjusted to 1 μg/μL.

For gene cloning, RNA from the *B. tabaci* MED whole body was used to prepare cDNA with a PrimeScript II First strand cDNA Synthesis Kit (Takara, Dalian, China). Gene-specific full-length primers (the primer pairs used are listed in Supplementary Table 3) were designed. The PCR was performed on an S1000 Thermal Cycler PCR System (Applied Biosystems, Foster City, CA, United States) using La Taq (Takara, Dalian, China). After amplification, bands of the expected size were purified and sequenced.

For qRT-PCR assays, each RNA was used to prepare cDNA with a PrimeScript RT Kit containing gDNA Eraser (Takara, Dalian, China). Gene-specific primers were then designed (the primer pairs used are listed in Supplementary Table 3). The qRT-PCR assay was performed on a QuantStudio 3 device (Applied Biosystems, Foster City, United States) using 2 × SuperReal PreMix Plus reagent (Tiangen, Beijing, China). *Elongation factor 1 alpha* (*EF1*α) was used as a reference gene (Li et al., 2013), and expression variation among samples was evaluated *via* the 2^–ΔΔ*Ct*^ method (Livak and Schmittgen, 2001).

### RNAi Construct and Gene Silencing

The RNAi constructs were generated and applied as previously described (Yang et al., 2016), and dsRNA for *enhanced green fluorescent protein* (*EGFP*) was used as a negative control. Before performing the RNAi assay, dsRNA for *BtTS*, *BtTK* and *EGFP* was prepared using a T7 RiboMAX Express RNAi system (Promega, Madison, United States) (the primers used are listed in Supplementary Table 3) and dissolved in 200 μL of artificial diet [100 μg of dsRNA, 5% yeast extract and 30% sucrose (wt/vol)]. The artificial diet was then fed to *B. tabaci* MED adults (mixture of both females and males) in feeding chambers. Each treatment involved six feeding chambers, and 70 newly emerged *B. tabaci* MED adults were introduced into each feeding chamber. The feeding chambers were then incubated in an environmental chamber at 25°C under a photoperiod of 14 L:10 D and a relative humidity (RH) of 70%.

### Determining EAA Biosynthesis Rates and *Portiera* Density

After silencing for 2 and 4 days, the mortality of *B. tabaci* MED adults was recorded. The surviving *B. tabaci* MED adults from each replication were then collected and divided into two groups. The first group contained 25 *B. tabaci* MED adults that were used to determine the RNAi silencing efficiency and the effects of silenced targeted genes on the expression of EAA biosynthesis-related genes. Thirty of the other surviving *B. tabaci* MED adults constituted the second group and were used to determine the *Portiera* density of each whitefly individual. Before determining the *Portiera* density of the individual insects, genomic DNA of each individual insect was extracted as previously described (Zheng et al., 2017). The density of *Portiera* in the *B. tabaci* MED adults was then assessed *via* a QuantStudio 3 device (Applied Biosystems, United States) using 2 × SuperReal PreMix Plus (Tiangen, Beijing, China). The protocol for the density of *Portiera* determination was the same as that described previously (Shan et al., 2016). The relative density of *Portiera* was quantified *via* their *16S rRNA* gene, while the β*-actin* gene (nuclear gene) of the whiteflies was used as an internal standard for normalization. All the primer pairs used are listed in Supplementary Table 3.

### Impact of *BtTS* and *BtTK* on Reproduction and Offspring Hatchability

To assess the effects of suppressing dTTP production on *B. tabaci* MED females, *BtTS* and *BtTK* of *B. tabaci* MED females were first knocked down *via* RNAi as described above. At 2 days after the induction of silencing, five *B. tabaci* MED females were released into one clip cages on health cotton plants. Three days after the release of the females, the number of eggs that were deposited on the leaves within the clip cages were counted. Later, egg hatchability was calculated. Each clip cage was considered one biological replicate, and each treatment had 20 replicates.

### Statistical Analysis

SPSS version 23.0 (SPSS Inc., Chicago, IL, United States) was used for statistical analysis. Differences among treatments were evaluated by one-way ANOVA in conjunction with Tukey’s test (*p* < 0.05).

## Results

### Genetic Capacity Analysis of the Pyrimidine Pathway in Selected Insects and Symbionts

**FIGURE 1:**
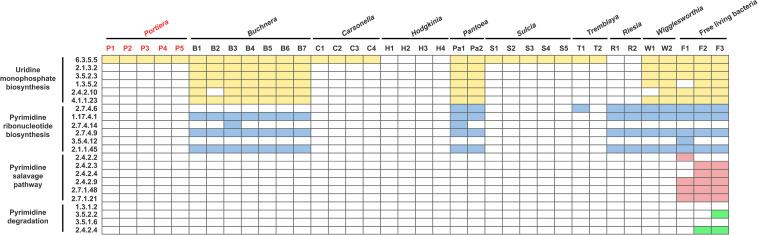
Content of genes involved in the pyrimidine pathway of obligate symbionts. The boxes representing genes involved in uridine monophosphate biosynthesis are colored yellow; pyrimidine ribonucleotide biosynthesis, blue; the pyrimidine salvage pathway, red; and pyrimidine degradation, green. The biosynthetic processes are shown in their reaction order. The EC number of each gene is also shown. For the obligate symbiont *Portiera*, the genes present are colored red. Detailed information on the select symbionts is listed in Supplementary Table 1.

**FIGURE 2:**
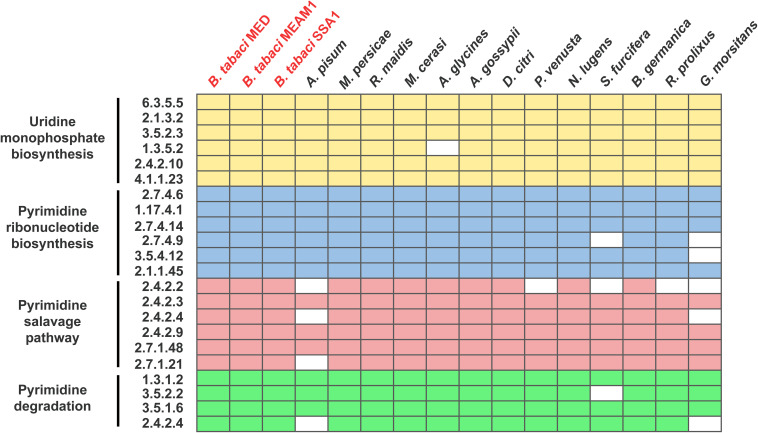
Content of genes involved in the pyrimidine pathway of host insects. The boxes representing genes involved in uridine monophosphate biosynthesis are colored yellow; pyrimidine ribonucleotide biosynthesis, blue; the pyrimidine salvage pathway, red; and pyrimidine degradation, green. Biosynthetic processes are shown in their linear order. The EC number of each gene is also shown. *B. tabaci* is colored red. Detailed information on the select insects is listed in Supplementary Table 2.

To ensure whether dTTP was a metabolic input that originated from host insects, genome-wide genetic capacity analysis of the pyrimidine pathway was performed. The results showed that *Portiera* encodes only one gene involved in the pyrimidine pathway (Figure 1), while *B. tabaci* MED encodes all the genes involved in the pyrimidine pathway (Figure 2). The results also showed that among selected obligate symbionts, only those of aphids (labeled *Buchnera*), stink bugs (labeled *Pantoea*) and tsetse flies (labeled *Wigglesworthia*) encode the whole set of genes for uridine monophosphate biosynthesis. No selected obligate symbiont was found to encode a complete set of genes for the pyrimidine salvage pathway and pyrimidine degradation (Figure 1). Though several obligate symbionts lacked the majority of pyrimidine pathway genes, their host insects still encoded them (Figure 2).

### Cloning, Phylogenetic Analysis and Expression Pattern Analysis

**FIGURE 3:**
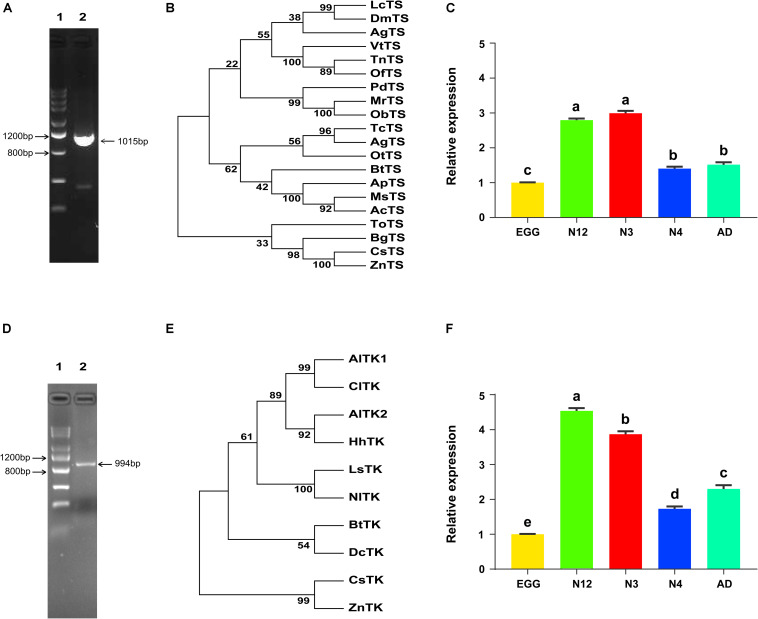
Cloning, phylogenetic analysis and expression patterns of both *BtTS* and *BtTK.* **(A,D)** Complete coding sequence amplification of *BtTS* and *BtTK* from MED. For panel **(A)**, line 1, Tiangen DNA marker III; line 2, *BtTS.* For panel **(D)**, line 1, Tiangen DNA marker III; line 2, *BtTK.* **(B,E)** Phylogenetic tree of *BtTS* and *BtTK* from MED. LcTS, *Lucilia cuprina*, XP_023302452.1; DmTS, *Drosophila melanogaster*, NP_001285570.1; AgTS, *Anopheles gambiae*, XP_311491.3; VtTS, *Vanessa tameamea*, XP_026491376.1; TnTS, *Trichoplusia ni*, XP_026747379.1; OfTS, *Ostrinia furnacalis*, XP_028163869.1; PdTS, *Polistes dominula*, XP_015173877.1; MrTS, *Megachile rotundata*, XP_003705109.1; ObTS, *Osmia bicornis*, XP_029051538.1; TcTS, *Tribolium castaneum*, XP_008191570.1; AgTS, *Anoplophora glabripennis*, XP_018568721.1; OtTS, *Onthophagus taurus*, XP_022918259.1; ApTS, *Acyrthosiphon pisum*, NP_001155666.1; MsTS, *Melanaphis sacchari*, XP_025205323.1; AcTS, *Aphis craccivora*, KAF0760635.1; FoTS, *Frankliniella occidentalis*, KAE8752504.1; BgTS, *Blattella germanica*, PSN45388.1; CsTS, *Cryptotermes secundus*, XP_023702879.1; ZnTS, *Zootermopsis nevadensis*, XP_021913580.1; AlTK1, *Apolygus lucorum*, KAE9428612.1; ClTK, *Cimex lectularius*, XP_014254368.1; AlTK2, *Apolygus lucorum*, KAE9423970.1; HhTK, *Halyomorpha halys*, XP_014279550.1; LsTK, *Laodelphax striatellus*, RZF40272.1; NlTK, *Nilaparvata lugens*, XP_022188850.1; DcTK, *Diaphorina citri*, XP_026679347.1; CsTK, *Cryptotermes secundus*, XP_023714579.1; ZnTK, *Zootermopsis nevadensis*, KDR10834.1. **(C,F)** Expression patterns of *BtTS* and *BtTK* across developmental stages. Egg, egg stage; N12, first and second nymph; N3, third nymph; N4. fourth nymph; AD, adult. The values shown are the means and standard errors, and the different letters indicate treatment differences at *p* < 0.05 (one-way ANOVA with Tukey’s test).

Before investigating the impacts of dTTP on *Portiera*, two important dTTP synthetic genes, *BtTS* and *BtTK*, were firstly cloned (Figures 3A,D). Phylogenetic analysis showed that *BtTS* and *BtTK* were relatively clustered with their homologous proteins in hemipteran insects (Figures 3B,E). The expression patterns of two genes showed that *BtTS* was most highly expressed in 3rd nymph stage while *BtTK* was most highly expressed in 1st and 2nd nymph stage (Figures 3C,F).

### Impact of *BtTS* and *BtTK* Silencing on EAA Biosynthesis and *Portiera* Density

**FIGURE 4:**
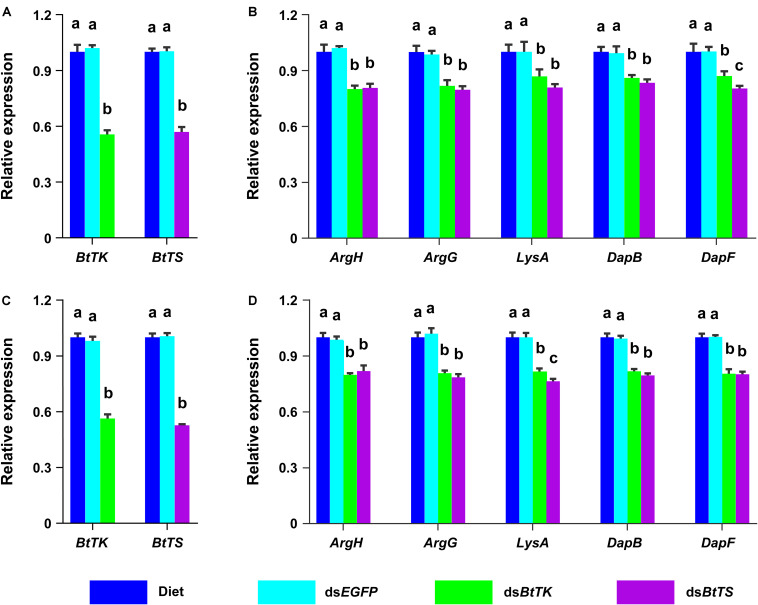
Temporal expression profiles of *BtTK*, *BtTS*, and EAA biosynthetic HTGs in *B. tabaci* MED treated with dsRNA or artificial diet. **(A)** Expression profiles of *BtTK* and *BtTS* in *B. tabaci* MED that had been treated for 2 days with RNAi constructs. **(B)** Expression profiles of EAA biosynthetic HTGs in *B. tabaci* MED after 2 days of treatment. **(C)** Expression profiles of *BtTK* and *BtTS* in *B. tabaci* MED after 4 days of treatment. **(D)** Expression profiles of EAA biosynthetic HTGs in *B. tabaci* MED after 4 days of treatment. For each gene, transcript levels in group of adults fed the artificial diet were normalized to one. The values shown are the means and standard errors, and the different letters indicate treatment differences at *p* < 0.05 (one-way ANOVA with Tukey’s test).

**FIGURE 5:**
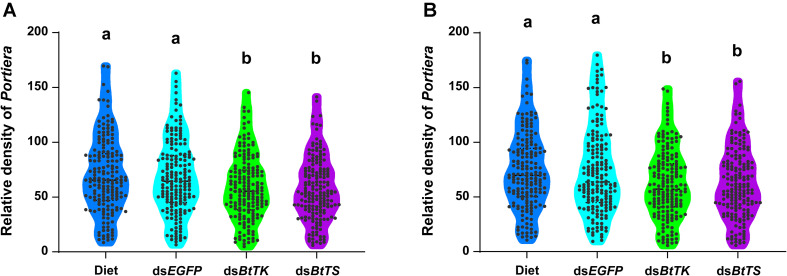
Temporal dynamics of *Portiera* in *B. tabaci* MED fed dsRNA or artificial diet. **(A)** Density of *Portiera* in *B. tabaci* MED after 2 days of treatment. **(B)** The density of *Portiera* in *B. tabaci* MED after 4 days of treatment. The changes in symbiont density were measured in terms of the number of *16S rRNA* gene copies per β*-actin* gene copy. The different letters indicate treatment differences at *p* < 0.05 (one-way ANOVA with Tukey’s test).

**FIGURE 6:**
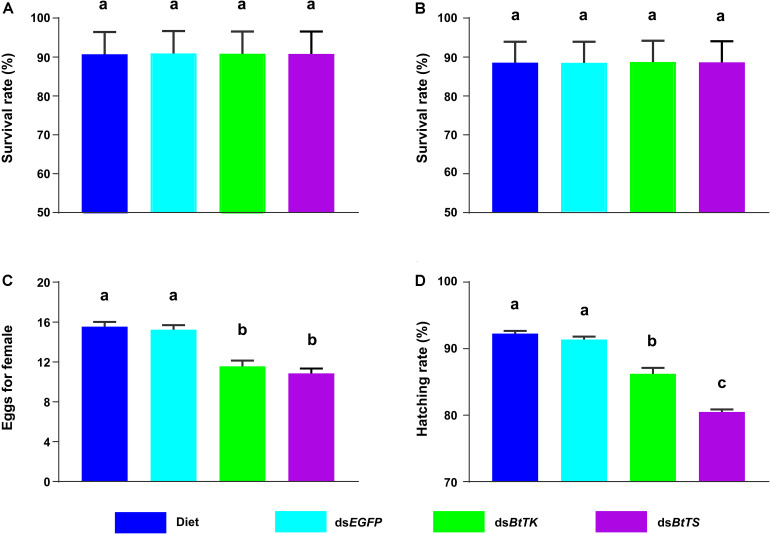
Effects of *BtTK* and *BtTS* silencing on *B. tabaci* MED. **(A)** Survival of *B. tabaci* MED adults upon treatment with dsRNA or artificial diet for 2 days. **(B)** Survival of *B. tabaci* MED adults after treatment with dsRNA or artificial diet for 4 days. **(C)** Effects of dsRNA or artificial diet on the egg production of *B. tabaci* females. **(D)** Effects of dsRNA or artificial diet on the hatchability of *B. tabaci* offspring. The values shown are the means and standard errors, and the different letters indicate treatment differences at *p* < 0.05 (one-way ANOVA with Tukey’s test).

To determine the effects of silencing *BtTS* and *BtTK* on *B. tabaci* MED adults and *Portiera*, RNAi constructs were applied to *B. tabaci* MED adults. The qRT-PCR results showed that upon silencing for 2 days (*BtTS*, *F*_2,15_ = 99.100, *p* < 0.0001; *BtTK*, *F*_2,15_ = 124.046, *p* < 0.0001) and 4 days (*BtTS*, *F*_2,15_ = 338.345, *p* < 0.0001; *BtTK*, *F*_2,15_ = 256.058, *p* < 0.0001), the expression levels of *BtTS* and *BtTK* significantly decreased (Figures 4A,C). Compared with control treatments in which *B. tabaci* MED adults were fed a normal diet or ds*EGFP*, feeding *B. tabaci* MED adults with ds*BtTS* and ds*BtTK* significantly decreased the expression levels of EAA biosynthesis-related genes (Figures 4B,D and Supplementary Table 4). For *BtTS*, silencing for 2 days (*F*_3,716_ = 7.236, *p* = 0.015) and 4 days (*F*_3,716_ = 5.880, *p* = 0.027) significantly reduced the density of *Portiera* (Figures 5A,B). The density of *Portiera* was also significantly reduced when *BtTK* was silenced for 2 days (*p* = 0.003) and 4 days (*p* = 0.033). Knocking down *BtTS* (2 days, *F*_3,20_ = 0.131, *p* = 0.999; 4 days, *F*_3,20_ = 0.144, *p* = 0.942) and *BtTK* (2 days, *p* = 0.989; 4 days, *p* = 0.992) did not cause a significant lethal effect (Figures 6A,B).

### Impact of *BtTS* and *BtTK* on Female Production and Offspring Hatchability

The effects of silencing *BtTS* and *BtTK* on the fitness of *B. tabaci* MED were also measured. Suppressing *BtTS* significantly decreased the reproduction of *B. tabaci* MED females (*F*_3,76_ = 23.986, *p* < 0.0001, Figure 6C) and the hatchability of *B. tabaci* MED offspring (*F*_3,76_ = 91.020, *p* < 0.0001, Figure 6D). Furthermore, suppressing *BtTK* also significantly decreased the reproduction of *B. tabaci* MED females (*p* < 0.0001, Figure 6C) and the hatchability of *B. tabaci* MED offspring (*p* < 0.0001, Figure 6D). Though no significant difference in the reproduction of *B. tabaci* MED females was observed between the ds*BtTS*-treated groups and ds*BtTK*-treated groups (*p* = 0.730), suppressing *BtTK* expression more significantly reduced hatchability than did suppressing *BtTS* expression (*p* < 0.0001; Figure 6D).

## Discussion

Here, a genetic capacity survey revealed that many obligate symbionts required their insects’ hosts to supply dTTP. Two dTTP synthesis-related genes, *BtTS* and *BtTK*, were then identified and cloned. Later, the influences of blocking dTTP production on *Portiera* and *B. tabaci* MED were investigated. Our results showed that suppressing dTTP production greatly repressed EAA biosynthesis, significantly decreased *Portiera* density and caused a serious decline in *B. tabaci* MED.

Because of genomic decay, obligate symbionts rely on their hosts to supply metabolic inputs for synthesizing essential nutrients (Moran et al., 2008; McCutcheon and Moran, 2011; Moran and Bennett, 2014). To date, host-supplied metabolic inputs have been largely reported in the EAA biosynthesis of obligate symbionts (as described in the introduction). Here, we found that many selected obligate symbionts such as *Portiera* were not able to produce dTTP, while their host insects retained such capacities. In view of the great demand for dTTP across whole life cycles, our study revealed that, except for EAA biosynthesis, dTTP is another kind of metabolic input in several insect/obligate symbiont symbiosis systems.

The metabolic input cystathionine was previously reported to determine methionine production in *A. pisum*/*Buchnera* symbiosis systems (Russell et al., 2014). It has also been proposed that metabolic inputs control obligate symbiont growth in insects (Ankrah et al., 2018). Here, our results showed that blocking dTTP production repressed the expression level of EAA synthetic HTGs and significantly reduced the density of *Portiera*. Given that those HTGs compensated for the gene loss of *Portiera* and that the majority of reactions of EAA biosynthesis were still afforded by *Portiera* (Luan et al., 2015; Xie et al., 2018), our observations indicated that suppressing *BtTS* and *BtTK* slowed the EAA biosynthesis of *Portiera*. Taken together, our results indicated that dTTP is applied as a regulator to control obligate symbionts in the *B. tabaci* MED/*Portiera* symbiosis system.

In addition, we also observed that blocking dTTP production significantly decreased the reproduction of *B. tabaci* MED adults and the hatchability of their offspring. The supply of enough EAAs is essential for insect reproduction (Roy et al., 2018; Toshima and Schleyer, 2019). EAAs also construct vitellogenin, which is a nutrient needed in egg hatching (Franz, 1979). As *Portiera* supplies EAAs for *B. tabaci* MED (Baumann, 2005; Xie et al., 2018), the negative effects we observed were likely caused by the fact that blocking dTTP production reduced the density of *Portiera* as well as EAA biosynthesis. As *B. tabaci* causes severe unfitness, *BtTS* and *BtTK* were suggested to be potential targets for symbiont-targeted *B. tabaci* MED management. Antibiotics have also been suggested to serve as novel pesticides for symbiont-targeted *B. tabaci* MED management (Zhang et al., 2015). Though also causing a series of negative effects on *B. tabaci* MED, the release of high concentrations of antibiotics was shown to take a heavy toll on the environment, such as influencing the structures and activities of microbes in the environment (Martinez, 2009). As silencing the two targeted genes blocked dTTP production only in *B. tabaci* MED and was not detrimental to environmental microbes, it seems that managing *B. tabaci* MED by silencing dTTP synthesis-related genes such as *BtTS* and *BtTK* is more friendly to the environment than managing *B. tabaci* MED by using antibiotics.

In addition to *B. tabaci* MED, other sucking insects such as psyllids and mealybugs are also important agricultural pests and harbor obligate symbionts (Baumann, 2005). Since obligate symbionts are essential for the survival of their hosts (Husnik et al., 2013; Sloan et al., 2014) and cannot be acquired from the environment (Koga et al., 2012; Luan et al., 2018), these obligate symbionts have been proposed as novel targets for pest control (Douglas, 2015). Here, we showed that the two dTTP synthesis-related genes that produce the dTTP that regulates the growth and metabolism of *Portiera* were potential targets for symbiont-targeted *B. tabaci* MED management. Interestingly, we also found that obligate symbionts of psyllids and mealybugs lack the capacities for biosynthesising dTTP. The results indicated that those symbionts may also require their hosts to supply dTTP. As dTTP is essential for cellular growth that is required throughout the whole life cycle, it is probable that genes involved in dTTP production could also serve as molecular targets for psyllid and mealy bug management.

In summary, we showed that suppressing dTTP production caused a series of negative effects on both *Portiera* and *B. tabaci* MED. Our results indicated that two dTTP synthetic genes, *BtTS* and *BtTK*, could be used as molecular targets for *B. tabaci* MED management. The study demonstrated a regulatory mechanism in the MED/*Portiera* system and likely revealed new molecular targets for whitefly pests and even management of other sucking insects.

## Data Availability Statement

All datasets presented in this study are included in the article/Supplementary Material.

## Author Contributions

ZY and YZ designed the research. YZ and CG conceived the experiments. YZ, YH, and JX analyzed the data. ZY and JZ drafted the manuscript. WX, ZG, XY, WX, SW, QW, and YZ revised and finalized the manuscript. All authors contributed to the article and approved the submitted version.

## Conflict of Interest

The authors declare that the research was conducted in the absence of any commercial or financial relationships that could be construed as a potential conflict of interest.
