# Supplementary material for: Two Deoxythymidine Triphosphate Synthesis-Related Genes Regulate Obligate Symbiont Density and Reproduction in the Whitefly Bemisia tabaci MED
Source: Front Physiol. 2021 Feb 24;11:574749. doi: 10.3389/fphys.2020.574749 (PMC7943623; doi:10.3389/fphys.2020.574749)
Supplement: Supplementary file 1 [file Table_1.DOCX]

**Supplementary Tables**

Table S1. Information of obligate symbiont and free-living bacteria selected in this study.

Table S2. Information of sucking insects selected in this study.

Table S3. Primers used in this study.

Table S4. Statistical analysis of EAAs biosynthetic genes when *BtKS* or *BtTK* was silenced.

Table S5. Pyrimidine pathway related genes of different *B. tabaci* cryptic specieses.

**Table S1**

| Label | Host insect | Full name | Obligate symbiont or Free living bacteria | Source |
| --- | --- | --- | --- | --- |
| P1 | Whitefly | *Candidatus Portiera aleyrodidarum* CAAS_CanPor1.0 | Obligate symbiont | https://www.ncbi.nlm.nih.gov/assembly/GCF_004300905.1 |
| P2 | Whitefly | *Candidatus Portiera aleyrodidarum* BT-B-HRs | Obligate symbiont | https://www.ncbi.nlm.nih.gov/assembly/GCF_000300075.1 |
| P3 | Whitefly | *Candidatus Portiera aleyrodidarum* BT-QVLC | Obligate symbiont | https://www.ncbi.nlm.nih.gov/assembly/GCF_000298385.1 |
| P4 | Whitefly | *Candidatus Portiera aleyrodidarum* MEAM1 | Obligate symbiont | ftp://www.whiteflygenomics.org/ |
| P5 | Whitefly | *Candidatus Portiera aleyrodidarum* TV-BCN | Obligate symbiont | https://www.ncbi.nlm.nih.gov/assembly/GCA_000953395.1 |
| B1 | Aphid | *Buchnera aphidicola* str. APS | Obligate symbiont | https://www.ncbi.nlm.nih.gov/assembly/GCF_000009605.1 |
| B2 | Aphid | *Buchnera aphidicola* str. G002 | Obligate symbiont | https://www.ncbi.nlm.nih.gov/assembly/GCF_000521565.1 |
| B3 | Aphid | *Buchnera aphidicola* str. Sg | Obligate symbiont | https://www.ncbi.nlm.nih.gov/assembly/GCF_000007365.1 |
| B4 | Aphid | *Buchnera aphidicola* str. 5A | Obligate symbiont | https://www.ncbi.nlm.nih.gov/assembly/GCF_000021085.1 |
| B5 | Aphid | *Buchnera aphidicola* str. Ak | Obligate symbiont | https://www.ncbi.nlm.nih.gov/assembly/GCF_000225445.1 |
| B6 | Aphid | *Buchnera aphidicola* str. USDA | Obligate symbiont | https://www.ncbi.nlm.nih.gov/assembly/GCF_000521525.1 |
| B7 | Aphid | *Buchnera aphidicola* str. W106 | Obligate symbiont | https://www.ncbi.nlm.nih.gov/assembly/GCF_000521545.1 |
| C1 | Psyllidae | *Candidatus Carsonella ruddii* HC isolate Thao2000 | Obligate symbiont | https://www.ncbi.nlm.nih.gov/assembly/GCF_000287275.1 |
| C2 | Psyllidae | *Candidatus Carsonella ruddii* HT isolate Thao2000 | Obligate symbiont | https://www.ncbi.nlm.nih.gov/assembly/GCF_000287295.1 |
| C3 | Psyllidae | *Candidatus Carsonella ruddii* BC | Obligate symbiont | https://www.ncbi.nlm.nih.gov/assembly/GCA_002009355.1 |
| C4 | Psyllidae | *Candidatus Carsonella ruddii* BT | Obligate symbiont | https://www.ncbi.nlm.nih.gov/assembly/GCF_002786955.1 |
| H1 | Cicadas | *Candidatus Hodgkinia cicadicola* Strain: MAGSDC | Obligate symbiont | https://www.ncbi.nlm.nih.gov/assembly/GCA_002774915.1 |
| H2 | Cicadas | *Candidatus Hodgkinia cicadicola* Strain: ALECUR | Obligate symbiont | https://www.ncbi.nlm.nih.gov/assembly/GCA_002762315.1 |
| H3 | Cicadas | *Candidatus Hodgkinia cicadicola* Strain: TRYCRA | Obligate symbiont | https://www.ncbi.nlm.nih.gov/assembly/GCA_002762275.1 |
| H4 | Cicadas | *Candidatus Hodgkinia cicadicola* Strain: MAGCAS | Obligate symbiont | https://www.ncbi.nlm.nih.gov/assembly/GCA_002762255.1 |
| Pa1 | Stink bug | *Candidatus Pantoea carbekii* | *Obligate symbiont* | https://www.ncbi.nlm.nih.gov/assembly/GCF_000478905.1 |
| Pa2 | Stink bug | *Candidatus Ishikawaella capsulata* Mpkobe | Obligate symbiont | https://www.ncbi.nlm.nih.gov/assembly/GCF_000828515.1 |
| S1 | Leafhopper | *Candidatus Sulcia muelleri* | *Obligate symbiont* | https://www.ncbi.nlm.nih.gov/assembly/GCF_003214365.1 |
| S2 | Leafhopper | *Candidatus Sulcia muelleri CARI* | *Obligate symbiont* | https://www.ncbi.nlm.nih.gov/assembly/GCF_000147035.1 |
| S3 | Leafhopper | *Candidatus Sulcia muelleri* str. Sulcia-ALF | Obligate symbiont | https://www.ncbi.nlm.nih.gov/assembly/GCF_000442635.1 |
| S4 | Leafhopper | *Candidatus Sulcia muelleri* DMIN | Obligate symbiont | https://www.ncbi.nlm.nih.gov/assembly/GCF_000025785.1 |
| S5 | Leafhopper | *Candidatus Sulcia muelleri* PSPU | Obligate symbiont | https://www.ncbi.nlm.nih.gov/assembly/GCF_000829155.1 |
| T1 | mealbug | *Candidatus Tremblaya princeps* PCIT | Obligate symbiont | https://www.ncbi.nlm.nih.gov/assembly/GCF_000219195.1 |
| T2 | mealbug | *Candidatus Tremblaya princeps* PCVAL | Obligate symbiont | https://www.ncbi.nlm.nih.gov/assembly/GCF_000220965.1 |
| R1 | Louse | *Candidatus Riesia pediculic1ola* USDA | Obligate symbiont | https://www.ncbi.nlm.nih.gov/assembly/GCF_000093065.1 |
| R2 | Louse | *Candidatus Riesia pediculicola* (enterobacteria) Strain: HHAN | Obligate symbiont | https://www.ncbi.nlm.nih.gov/assembly/GCA_002073935.1 |
| W1 | Tsetse fly | *Wigglesworthia glossinidia* endosymbiont of *Glossina brevipalpis* | Obligate symbiont | https://www.ncbi.nlm.nih.gov/assembly/GCF_000008885.1 |
| W2 | Tsetse fly | *Wigglesworthia glossinidia* endosymbiont of *Glossina morsitans* | Obligate symbiont | https://www.ncbi.nlm.nih.gov/assembly/GCF_000247565.1 |
| F1 | None | *Bacillus subtilis* subsp. subtilis 168 | Free living bacteria | KEGG |
| F2 | None | *Enterobacter cloacae* subsp. cloacae ATCC 13047 | Free living bacteria | KEGG |
| F3 | None | *Escherichia coli* K-12 MG1655 | Free living bacteria | KEGG |

**Table S2**

| **Full name** | **Source** |
| --- | --- |
| *B. tabaci* MED | ftp://www.whiteflygenomics.org/ |
| *B. tabaci* MEAM1 | ftp://www.whiteflygenomics.org/ |
| *B. tabaci* SSA1 | ftp://www.whiteflygenomics.org/ |
| *A. pisum* | https://www.ncbi.nlm.nih.gov/assembly/GCF_005508785.1 |
| *M. persicae* | https://bipaa.genouest.org/data/group/myzus/ |
| *R. maidis* | https://bipaa.genouest.org/sp/myzus_persicae/ |
| *M. cerasi* | https://bipaa.genouest.org/sp/myzus_cerasi/ |
| *A. glycines* | https://bipaa.genouest.org/sp/aphis_glycines/ |
| *A. gossypii* | https://www.ncbi.nlm.nih.gov/assembly/GCF_004010815.1 |
| *D. citri* | https://www.ncbi.nlm.nih.gov/assembly/GCF_000475195.1/ |
| *P. venusta* | https://i5k.nal.usda.gov/ |
| *N. lugens* | https://www.ncbi.nlm.nih.gov/assembly/GCF_000757685.1/ |
| *S. furcifera* | http://gigadb.org/dataset/100255 |
| *B. germanica* | https://i5k.nal.usda.gov/ |
| *R. prolixus* | https://www.vectorbase.org/downloads |
| *G. morsitans* | https://www.vectorbase.org/downloads |

**Table S3**

| **Primer** | **Gene/Application** | **Sequences (5'-3')** |
| --- | --- | --- |
| BtTSF | *BtTS*/Cloning | CCAAGTTAAAGGATTGTGTCT |
| BtTSR | *BtTS*/Cloning | CAATCAATAAAAGGCAAAGG |
| BtTKF | *BtTK*/Cloning | AATCGAAAATCATAGTGTTTGGCG |
| BtTKR | BtTK/Cloning | AAAGGTGGGAAAAGAGCAAGGTA |
| qBtTSF | *BtTS*/qRT-PCR | GACTTGGGTCCTGTTTATGGC |
| qBtTSR | *BtTS*/qRT-PCR | GAAGTTGTCGTTTGAGCTGCA |
| qBtTKF | *BtTK*/qRT-PCR | AGGTTGAAGTGATCGGAG |
| qBtTKR | *BtTK*/qRT-PCR | TATTTACCTTATTCCATCTTACA |
| qArgHF | *ArgH*/qRT-PCR | CATCGGCATGGTCCACCTCT |
| qArgHR | *ArgH*/qRT-PCR | TCTTCTTCTGCGGCATCAGC |
| qArgGF | *ArgG*/qRT-PCR | ACCTTACGGGTGTTTGGAGA |
| qArgGR | *ArgG*/qRT-PCR | GACTTAATGCCCACCAACCT |
| qLysAF | *LysA*/qRT-PCR | CTGTTGGTCAATGGATCAGG |
| qLysAR | *LysA*/qRT-PCR | AGCTCCTCGGCAATTAAGTC |
| qDapFF | *DapF*/qRT-PCR | GCATGGTCCTCTCCATCAC |
| qDapFR | *DapF*/qRT-PCR | ACCGAATGTCACGTTATCCA |
| qDapBF | *DapB*/qRT-PCR | CACCACCGGATGAAGTTAGA |
| qDapBR | *DapB*/qRT-PCR | CAATTTCGCCCGATAGTCTC |
| qBtF | *β-actin*/density | TCTTCCAGCCATCCTTCTTG |
| qBtR | *β-actin*/density | CGGTGATTTCCTTCTGCATT |
| qPorF | *16S/*density | GTGGGGAATAACGTACGG |
| qPorF | *16S/*density | CTCAGTCCCAGTGTGGCTG |
| rBtTSF | *BtTS*/RNAi | TAATACGACTCACTATAGGGAGAGACTTGGGTCCTGTTTATGGC |
| rBtTSR | *BtTS*/ RNAi | TAATACGACTCACTATAGGGAGAGAAGTTGTCGTTTGAGCTGCA |
| rBtTKF | *BtTK*/RNAi | TAATACGACTCACTATAGGGAGAAGGTTGAAGTGATCGGAG |
| rBtTKR | *BtTK*/RNAi | TAATACGACTCACTATAGGGAGATATTTACCTTATTCCATCTTACA |
| rEGFPF | *EGFP*/RNAi | TAATACGACTCACTATAGGGAGACAGTGCTTCAGCCGCTAC |
| rEGFPR | *EGFP*/RNAi | TAATACGACTCACTATAGGGAGAAGTTCACCTTGATGCCGTTC |

**Table S4.**

| Days | 2 days | | 4 days | |
| --- | --- | --- | --- | --- |
|  | ds*BtTS* | ds*BtTK* | ds*BtTS* | ds*BtTK* |
| *ArgH* | F_3,20_=207.50; *p* < 0.0001 | F_3,20_=207.50; *p* < 0.0001 | F_3,20_=290.499; *p* < 0.0001 | F_3,20_=290.499; *p* < 0.0001 |
| *ArgG* | F_3,20_=148.549; *p* < 0.0001 | F_3,20_=148.549; *p* < 0.0001 | F_3,20_=261.287; *p* < 0.0001 | F_3,20_=261.287; *p* < 0.0001 |
| *LysA* | F_3,20_=79.414; *p* < 0.0001 | F_3,20_=79.414; *p* < 0.0001 | F_3,20_=238.152; *p* < 0.0001 | F_3,20_=238.152; *p* < 0.0001 |
| *DapB* | F_3,20_=97.426; *p* < 0.0001 | F_3,20_=97.426; *p* < 0.0001 | F_3,20_=456.301; *p* < 0.0001 | F_3,20_=456.301; *p* < 0.0001 |
| *DapF* | F_3,20_=65.836; *p* < 0.0001 | F_3,20_=65.836; *p* < 0.0001 | F_3,20_=411.102; *p* < 0.0001 | F_3,20_=411.102; *p* < 0.0001 |

**Table S5.**

| **Gene** | c**ryptic species** | **KO number** | **EC number** |
| --- | --- | --- | --- |
| BTA008028.1 | *B. tabaci* MED | K11540 | 6.3.5.5, 2.1.3.2, 3.5.2.3 |
| BTA013708.1 | *B. tabaci* MED | K00254 | 1.3.5.2 |
| BTA018328.1 | *B. tabaci* MED | K00254 | 1.3.5.2 |
| BTA014276.1 | *B. tabaci* MED | K13421 | 2.4.2.10, 4.1.1.23 |
| BTA027970.1 | *B. tabaci* MED | K00940 | 2.7.4.6 |
| BTA002013.1 | *B. tabaci* MED | K00940 | 2.7.4.6 |
| BTA006332.1 | *B. tabaci* MED | K00940 | 2.7.4.6 |
| BTA002310.1 | *B. tabaci* MED | K10807 | 1.17.4.1 |
| BTA002382.1 | *B. tabaci* MED | K10808 | 1.17.4.1 |
| BTA004572.1 | *B. tabaci* MED | K00943 | 2.7.4.9 |
| BtQcomEB1^a^ | *B. tabaci* MED | K01493 | 3.5.4.12 |
| BtQcomEB1^b^ | *B. tabaci* MED | K01493 | 3.5.4.12 |
| BTA019457.2 | *B. tabaci* MED | K00560 | 2.1.1.45 |
| BTA019071.1 | *B. tabaci* MED | K00757 | 2.4.2.3 |
| BTA009830.1 | *B. tabaci* MED | K00757 | 2.4.2.3 |
| BTA026439.1 | *B. tabaci* MED | K00758 | 2.4.2.4 |
| BTA027846.1 | *B. tabaci* MED | K00761 | 2.4.2.9 |
| BTA018497.1 | *B. tabaci* MED | K00857 | 2.7.1.21 |
| BTA009163.1 | *B. tabaci* MED | K00876 | 2.7.1.48 |
| BTA015301.1 | *B. tabaci* MED | K00876 | 2.7.1.48 |
| BTA002491.1 | *B. tabaci* MED | K00207 | 1.3.1.2 |
| BTA001710.1 | *B. tabaci* MED | K01431 | 3.5.1.6 |
| BTA007456.1 | *B. tabaci* MED | K01431 | 3.5.1.6 |
| BTA026439.1 | *B. tabaci* MED | K00758 | 2.4.2.4 |
| Bta12325 | *B. tabaci* MEAM1 | K11540 | 6.3.5.5, 2.1.3.2, 3.5.2.3 |
| Bta07905 | *B. tabaci* MEAM1 | K00254 | 1.3.5.2 |
| Bta01575 | *B. tabaci* MEAM1 | K13421 | 2.4.2.10, 4.1.1.23 |
| Bta08348 | *B. tabaci* MEAM1 | K00940 | 2.7.4.6 |
| Bta02556 | *B. tabaci* MEAM1 | K10807 | 1.17.4.1 |
| Bta11444 | *B. tabaci* MEAM1 | K10808 | 1.17.4.1 |
| Bta00886 | *B. tabaci* MEAM1 | K00943 | 2.7.4.9 |
| Bta01900 | *B. tabaci* MEAM1 | K01493 | 3.5.4.12 |
| Bta01901 | *B. tabaci* MEAM1 | K01493 | 3.5.4.12 |
| Bta09205 | *B. tabaci* MEAM1 | K00560 | 2.1.1.45 |
| Bta13218 | *B. tabaci* MEAM1 | K00757 | 2.4.2.3 |
| Bta08411 | *B. tabaci* MEAM1 | K00758 | 2.4.2.4 |
| Bta07777 | *B. tabaci* MEAM1 | K00761 | 2.4.2.9 |
| Bta07777 | *B. tabaci* MEAM1 | K00857 | 2.7.1.21 |
| Bta05015 | *B. tabaci* MEAM1 | K00876 | 2.7.1.48 |
| Bta05015 | *B. tabaci* MEAM1 | K00876 | 2.7.1.48 |
| Bta06996 | *B. tabaci* MEAM1 | K00207 | 1.3.1.2 |
| Bta09069 | *B. tabaci* MEAM1 | K00207 | 3.5.1.6 |
| Bta08411 | *B. tabaci* MEAM1 | K00758 | 2.4.2.4 |
| Ssa03115 | *B. tabaci* SSA1 | K11540 | 6.3.5.5, 2.1.3.2, 3.5.2.3 |
| Ssa09651 | *B. tabaci* SSA1 | K00254 | 1.3.5.2 |
| Ssa05884 | *B. tabaci* SSA1 | K13421 | 2.4.2.10, 4.1.1.23 |
| Ssa08708 | *B. tabaci* SSA1 | K00940 | 2.7.4.6 |
| Ssa12621 | *B. tabaci* SSA1 | K10807 | 1.17.4.1 |
| Ssa14278 | *B. tabaci* SSA1 | K10808 | 1.17.4.1 |
| Ssa14353 | *B. tabaci* SSA1 | K10808 | 1.17.4.1 |
| Ssa08043 | *B. tabaci* SSA1 | K00943 | 2.7.4.9 |
| Ssa10185 | *B. tabaci* SSA1 | K01493 | 3.5.4.12 |
| Ssa00432 | *B. tabaci* SSA1 | K00560 | 2.1.1.45 |
| Ssa15120 | *B. tabaci* SSA1 | K00757 | 2.4.2.3 |
| Ssa04318 | *B. tabaci* SSA1 | K00758 | 2.4.2.4 |
| Ssa07399 | *B. tabaci* SSA1 | K00761 | 2.4.2.9 |
| Ssa00559 | *B. tabaci* SSA1 | K00857 | 2.7.1.21 |
| Ssa08573 | *B. tabaci* SSA1 | K00857 | 2.7.1.21 |
| Ssa13298 | *B. tabaci* SSA1 | K00876 | 2.7.1.48 |
| Ssa11193 | *B. tabaci* SSA1 | K00876 | 2.7.1.48 |
| Ssa08308 | *B. tabaci* SSA1 | K00207 | 1.3.1.2 |
| Ssa05646 | *B. tabaci* SSA1 | K01431 | 3.5.1.6 |
| Ssa04318 | *B. tabaci* SSA1 | K00758 | 2.4.2.4 |

^a^ BtQcomEB1 was unannotated in the current version of *B. tabaci* MED. BtQcomEB1 located on Scaffold22 from 1025804 to 1027599. ^b^ BtQcomEB2 was unannotated in the current version of *B. tabaci* MED. BtQcomEB2 located on Scaffold1044 from 234081 to 235885.
